# Polymorphisms and a Haplotype in Heparanase Gene Associations with the Progression and Prognosis of Gastric Cancer in a Northern Chinese Population

**DOI:** 10.1371/journal.pone.0030277

**Published:** 2012-01-20

**Authors:** Ai-Lin Li, Yong-Xi Song, Zhen-Ning Wang, Peng Gao, Yuan Miao, Jin-Liang Zhu, Zhen-Yu Yue, Hui-Mian Xu

**Affiliations:** 1 Department of Surgical Oncology and General Surgery, The First Hospital of China Medical University, Shenyang, People's Republic of China; 2 Department of Radiotherapy, The First Hospital of China Medical University, Shenyang, People's Republic of China; 3 Department of Pathology, The First Hospital of China Medical University, Shenyang, People's Republic of China; National Cancer Center, Japan

## Abstract

**Background:**

Human heparanase plays an important role in cancer development and single nucleotide polymorphisms (SNPs) in the heparanase gene (HPSE) have been shown to be correlated with gastric cancer. The present study examined the associations between individual SNPs or haplotypes in HPSE and susceptibility, clinicopathological parameters and prognosis of gastric cancer in a large sample of the Han population in northern China.

**Methodology/Principal Findings:**

Genomic DNA was extracted from formalin-fixed, paraffin-embedded normal gastric tissue samples from 404 patients and from blood from 404 healthy controls. Six SNPs were genotyped by matrix-assisted laser desorption/ionization time-of-flight mass spectrometry. A chi-square (χ2) test and unconditional logistic regression were used to analyze the risk of gastric cancer; a Log-rank test and Cox proportional hazards model were used to produce survival analysis and a Kaplan-Meier method was used to map survival curves. The mean genotyping success rates were more than 99% in both groups. Haplotype CA in the block composed of rs11099592 and rs4693608 had a greater distribution in the group of Borrmann types 3 and 4 (P = 0.037), the group of a greater number of lymph node metastases (N3 vs N0 group, P = 0.046), and moreover was correlated to poor survival (CG vs CA: HR = 0.645, 95%CI: 0.421–0.989, P = 0.044). In addition, genotypes rs4693608 AA and rs4364254 TT were associated with poor survival (P = 0.030, HR = 1.527, 95%CI: 1.042–2.238 for rs4693608 AA; P = 0.013, HR = 1.546, 95%CI: 1.096–2.181 for rs4364254 TT). There were no correlations between individual SNPs or haplotypes and gastric cancer risk.

**Conclusions/Significance:**

A functional haplotype in HPSE was found, which included the important SNP rs4693608. SNPs in HPSE play an important role in gastric cancer progression and survival, and perhaps may be a molecular marker for prognosis and treatment values.

## Introduction

Gastric cancer is the fourth most common cancer worldwide and second leading cause of cancer mortality [1]. Despite advances in diagnosis and treatment, the prognosis for patients with advanced gastric cancer remains dismal [2]. Furthermore, gastric cancer is a disease of gene-environment interactions and genetic factors play an important role in tumorigenesis and progression [3]. Therefore, discovery and application of biomarkers incorporated with traditional cancer diagnosis, staging, and prognosis could be considered the best option for controlling this life-threatening disease [4].

Single nucleotide polymorphisms (SNPs) have been thought to be attractive biomarkers in cancer risk assessment, screening, staging, or grading [5]. Also, the human genome is composed of a series of ‘haplotype blocks’, which are nonrandom associations of alleles due to linkage disequilibrium (LD) and it is possible to exploit a vast amount of information considering these haplotype blocks [6], [7]. Although the application of individual SNP analysis has been limited thus far, haplotype-based association study has been proposed as a powerful and comprehensive approach to identify causal genetic variation underlying complex diseases [8], [9].

Heparanase is the only known mammalian enzyme that degrades heparan sulfate (HS) proteoglycans in basement membranes and the extracellular matrix [10]. This leads to disassembly of extracellular barriers, release of HS-bound bioactive factors and generation of HS fragments that promote growth factor-receptor binding and signaling [11], [12]. Heparanase is strongly associated with cancer progression and metastasis, including cell survival, invasion, proliferation, neovascularization, and the creation of a growth-permissive microenvironment [13], [14] and it has both prognostic and therapeutic applications [15]. The heparanase gene (HPSE), first cloned in 1999, is located on chromosome 4q21.3 [16]. There have been few studies on SNPs in the HPSE gene. Molecular epidemiologic studies have shown distribution differences in SNPs in HPSE in various Israeli Jewish populations [17]. Associations to tumor susceptibility have also been demonstrated, including hematological malignancies and gastric cancer, but the results have not been accordant [18]–[20]. In addition, Shirley Ralphand [21] has shown an HPSE haplotype was correlated to stages in ovarian carcinoma and Yue et al. [20] have shown SNPs were correlated to clinicopathological parameters and survival rate. Specifically, the study indicated that SNPs in HPSE were associated with heparanase expression levels and provided the basis for further studies on the associations between SNPs and disease [22]. However, these association studies were limited to small samples.

Recently, Hennig G [23] and Horn H [24] observed high genotyping detection rates (93.5% and 94–97%) and a perfect concordance rate of 100% with DNA extracted from normal formalin-fixed, paraffin-embedded tissues (FFPETs) compared to germline DNA using matrix-assisted laser desorption/ionization time-of-flight mass spectrometry (MALDI-TOF MS). Besides, other reports also demonstrated high genotyping detection rates and a perfect concordance rate with FFPET-derived DNA including decades-old blocks compared to blood from the same individual using other methods, even in genome-wide genotyping [25]–[28]. It has been ascertained that FFPET-derived DNA was sufficient for genetic polymorphism analysis. In the present study, we used a large collection of FFPET-derived DNA samples from patients and blood-derived DNA from controls in a MALDI-TOF MS method to genotype and study the potential associations between six SNPs (rs4693602, rs6856901, rs4364254, rs11099592, rs4693608 and rs4328905) or haplotypes in HPSE and tumor susceptibility, clinicopathological parameters, and survival of gastric cancer with a large sample of the Han population in northern China. As a result, individual SNPs and a haplotype were found to show associations with the progression and prognosis of gastric cancer.

## Results

### Subject characteristics

The average age was 56.67±11.923 y and the percentage of males was 70.54% in the case group. The average age of the control group was 56.91±11.477 y and the percentage of males was 70.54%. There was no distribution difference in sex and age between the patients and controls (P = 1.00 for both sex and age). Of the 404 patients, stage I gastric cancer cases accounted for 21.0% (85/404), stage II gastric cancer cases accounted for 26.5% (107/404) and stage III gastric cancer cases accounted for 52.5% (212/404) (Table 1).

**Table 1:** Distributions of selected characteristics in gastric cancer cases and controls (n = 404 for both case and control groups).

Variable	Patients (n = 404)No. (%)	Controls (n = 404)No. (%)	P*
Sex			
Male	285(70.54)	285(70.54)	1.00
Female	119(29.46)	119(29.46)	
Age at diagnosis			
≤40	30 (7.43)	30 (7.43)	1.00
41–50	94(23.27)	94(23.27)	
51–60	111(27.48)	111(27.48)	
61–70	122(30.20)	122(30.20)	
>70	47(11.63)	47(11.63)	
Tumor stage at diagnosis			
Ia	45(11.1)		
Ib	40(9.9)		
IIa	58(14.4)		
IIb	49(12.1)		
IIIa	57(14.1)		
IIIb	115(28.5)		
IIIc	40(9.9)		
IV	0(0)		

*Two-sided χ^2^ test.

### Genotyping success rates

We used MassArray Typer Analyzer software 4.0.4.20 for automated spectra processing and genotype identification. Representative MALDI-TOF-MS profiles of each genotype of the six SNPs in HPSE were shown in Figure S1. All the SNPs were polymorphic with minor allele frequency >10% and genotype distributions were all in agreement with Hardy-Weinberg equilibrium (data not shown). High success rates, ranging between 96.29% and 100% (mean: 99.09%) in the FFPETs group and between 99.50% and 100% (mean: 99.79%) in control group, were shown (Table S1).

### Associations between individual SNPs and clinicopathological parameters and survival

Allelic frequencies and genotypic frequencies in the six SNPs were not significantly different between patients and controls (P>0.05 and P>0.05 after a permutation test for allelic frequencies; P>0.05 and P>0.05 after being adjusted for sex and age for genotypic frequencies; Tables S2 and S3).

SNPs were evaluated for associations with the clinicopathological parameters. rs4364254 genotypes were associated with histologic grades (P = 0.002; Table S4), genotype TT was correlated to well cell differentiation compared to the genotype TC/CC (OR = 0.482; 95%CI: 0.300–0.774). Other SNPs had no significant correlations to clinicopathological parameters.

**Figure 1:**
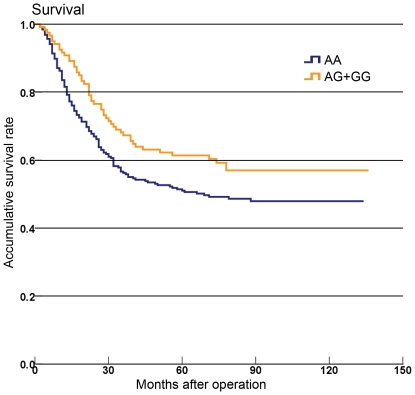
Kaplan-Meier survival curve analysis with rs4693608 genotypes. Results show that accumulative survival rate of 381 cases with gastric cancer were associated with the different rs4693608 genotypes in HPSE gene.

In univariate analysis, patients carrying the rs4693608 AA genotype had poor gastric cancer–specific survival compared to patients with the AG+GG genotype (P = 0.049, HR = 1.387, 95%CI: 1.001–1.923; Table 2, Figure 1 and Figure S2). In multivariate analysis, the rs4693608 AA genotype and rs4364254 TT genotype both had a poor gastric cancer–specific survival (P = 0.030, HR = 1.527, 95%CI: 1.042–2.238 for rs4693608; P = 0.013, HR = 1.546, 95%CI: 1.096–2.181 for rs4364254; Table 2). Also, Borrmann type (P<0.001), pT category (P<0.001), pN category(P<0.001), and lymphovascular invasion (P<0.001) were significantly correlated with survival in univariate analysis. Borrmann type (P = 0.021), pT category (P<0.001), and pN category (P<0.001) remained significantly correlated with survival in multivariate analysis (Table 2).

**Table 2:** Univariate and multivariate Cox proportional hazard analysis for gastric cancer patients of the six SNPs in HPSE Versus clinicopathological parameters (n = 381).

		*Univariate*			*Multivariate*		
Parameter		HR	95%CI	P	HR	95%CI	P
Sex	Female vs Male	1.138	0.831–1.560	0.420			
Age		1.006	0.992–1.019	0.418			
pT category		2.289	1.897–2.762	**<0.001** *	1.633	1.305–2.044	**<0.001** *
pN category		2.097	1.813–2.425	**<0.001** *	1.756	1.494–2.062	**<0.001** *
Borrmann type	Borr1+2vsBorr3+4	4.283	2.688–6.823	**<0.001** *	1.768	1.099–2.871	**0.021** *
Histologic grade	well vs poor	1.147	0.807–1.629	0.445			
Venous invasion		1.851	0.591–5.793	0.290			
Lymphovascular invasion		2.015	1.478–2.748	**<0.001** *			
rs4693602	GG vs GA+AA	1.293	0.917–1.825	0.143			
rs6856901	CC vs CG+GG	1.294	0.908–1.845	0.153			
rs4364254	TT vs TC+CC	1.025	0.766–1.371	0.868	1.546	1.096–2.181	**0.013** *
rs11099592	CC vs CT+TT	1.128	0.770–1.652	0.537			
rs4693608	AA vs AG+GG	1.387	1.001–1.923	**0.049** *	1.527	1.042–2.238	**0.030** *
rs4328905	AA vs AG+GG	1.052	0.775–1.427	0.747			

*Statistically significant (P<0.05).

Abbreviation: HR, hazard rate; CI, confidence interval.

### Presence of a haplotype related to clinicopathologic features and survival

**Figure 2:**
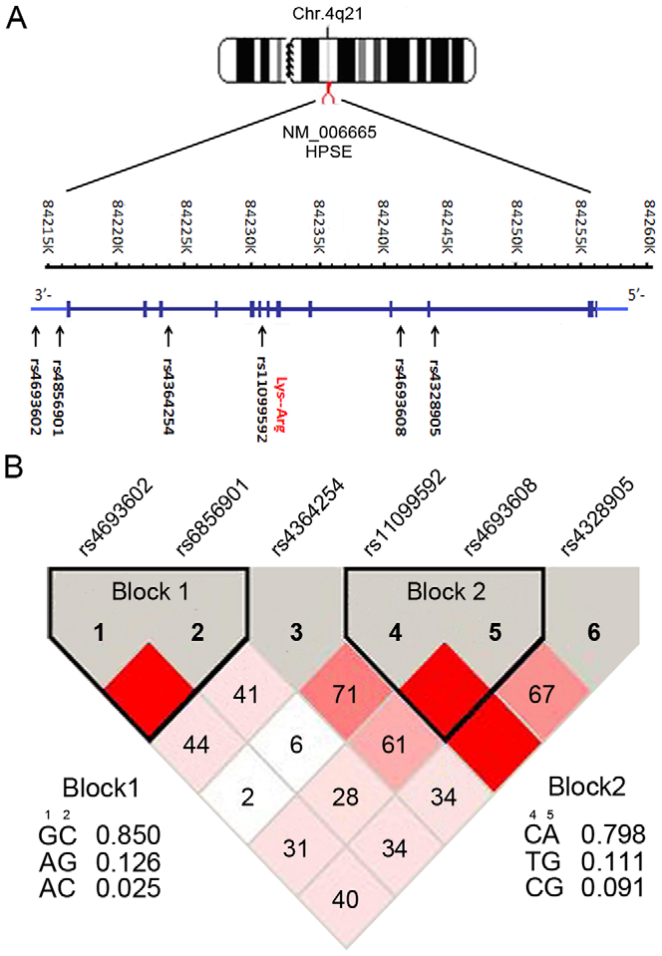
SNPs in the region of the HPSE gene cluster located on chromosome 4q21. A: HPSE gene structure. Filled boxes represent the 13 exons (5′→3′). Arrows show the locations of SNPs. B: Mapping of the block structure of the six SNPs generated by Haploview. The value within each square in the triangle plot represents the pairwise correlation between SNPs (measured as D') defined by the upper-left and the upper-right sides of the Squares. The Squares without a number correspond to D' = 1. Shading represents the magnitude and significance of pairwise LD, with a red-to-white gradient reflecting higher to lower LD values. The frequency of each common haplotype within a block is to the side of the haplotype.

There were two two-marker haplotype blocks constructed between the six SNPs in our results (Figure 2). Block 1 was composed of rs4693602 and rs6856901 and contained three common haplotypes (frequency range: 0.025–0.850), which represented approximately 99.9% of the subjects; block 2 was composed of rs11099592 and rs4693608 and also contained three common haplotypes (frequency range: 0.091–0.798), which represented approximately 99.9% of the subjects. All six common haplotypes had no correlation with gastric cancer risk (P>0.05 and P>0.05 after a permutation test; Table S5).

Associations between haplotypes in HPSE and gastric cancer clinicopathologic features at the time of diagnosis were evaluated. Haplotype CA in block 2 had greater distribution in the group of Borrmann types 3 and 4 compared to TG+CG haplotypes (P = 0.037; Table 3). Haplotype distribution differences were observed in the pN category (P = 0.045; Table 3), CA had a greater distribution in the N3 group than the N0 group compared to CG (OR = 1.837,95%CI: 1.010–3.341, P = 0.046), but there were no significant distribution differences between the N2 and N0 groups and between the N1 and N0 groups (P = 0.671 and P = 0.496, respectively).

**Table 3:** Associations between haplotype frequencies of the six SNPs in HPSE and clinicopathological parameters (n = 404).

	Block1				Block2			
	GC	AG	AC	overall	CA	TG	CG	overall
Borrmann type								
Borr1+2	0.822	0.154	0.023		0.769	0.125	0.106	
Borr3+4	0.853	0.122	0.025		0.834	0.099	0.067	
P*	0.445	0.377	0.895	0.673	**0.037** #	0.29	0.076	0.095
Histologic grade								
Well	0.839	0.122	0.039		0.800	0.116	0.084	
Poor	0.846	0.134	0.020		0.822	0.103	0.075	
P*	0.579	0.975	0.144	0.343	0.430	0.544	0.659	0.731
pT category								
T1	0.789	0.175	0.035		0.763	0.136	0.102	
T2	0.873	0.112	0.015		0.807	0.121	0.071	
T3	0.841	0.126	0.033		0.824	0.097	0.079	
T4	0.864	0.130	0.006		0.848	0.091	0.061	
P*	0.363	0.575	0.344	0.539	0.069	0.160	0.329	0.189
pN category								
N0	0.795	0.176	0.029		0.808	0.096	0.096	
N1	0.894	0.074	0.032		0.796	0.092	0.112	
N2	0.855	0.122	0.023		0.809	0.118	0.073	
N3	0.864	0.117	0.019		0.835	0.114	0.051	
P*	0.108	0.158	0.444	0.272	0.445	0.427	**0.045** #	0.114
Venous invasion								
Negative	0.845	0.131	0.025		0.815	0.107	0.078	
Positive	0.833	0.167	0.000		1.00	0.000	0.000	
P*	0.464	0.340	0.674	0.586	0.181	0.327	0.423	0.409
Lymphovascular invasion								
Negative	0.826	0.141	0.026		0.821	0.096	0.076	
Positive	0.879	0.101	0.020		0.785	0.135	0.080	
P*	0.090	0.101	0.663	0.232	0.187	0.125	0.872	0.293
TNM stage								
I	0.829	0.146	0.024		0.806	0.100	0.094	
II	0.822	0.139	0.038		0.792	0.108	0.099	
III	0.862	0.121	0.017		0.833	0.107	0.059	
P*	0.267	0.443	0.320	0.451	0.259	0.873	0.076	0.207

*Two-sided χ^2^ test, each haplotype compared with all other haplotypes.

# Statistically significant (P<0.05).

The haplotypes of block 2 indicated a significant difference in tumor-related survival. Patients carrying the CA haplotype had a poor gastric cancer–specific survival (CG vs CA: HR = 0.645, 95%CI: 0.421–0.989, P = 0.044; Table 4).

**Table 4:** Survival analysis of haplotypes of the six SNPs in HPSE (n = 381).

	Frequencies	HR(95%CI)	P*
Block1			
GC	0.846	1	
AG	0.022	0.847(0.627–1.146)	0.281
AC	0.132	0.676(0.299–1.528)	0.346
Block2			
CA	0.823	1	
TG	0.101	0.830(0.576–1.196)	0.317
CG	0.077	0.645(0.421–0.989)	**0.044**

*Based on Cox proportional hazards survival regression in haplotype-based association analysis using the Stochastic-EM algorithm.

Abbreviation: HR, hazard rate; CI, confidence interval.

## Discussion

As we have known, FFPET-derived DNA has lower extraction efficacy and quality (fragmented DNA) due to partial nucleic acid cross-linking and degradation than blood-derived DNA. But archived FFPETs provide an invaluable source for molecular genetic studies with several advantages such as (i) the only type of samples available for individuals who cannot otherwise provide a DNA sample, (ii) an excellent resource for large-scale retrospective biomarker studies, (iii) a large number of samples conjunct with long-term clinical follow-up data, (iv) a valuable resource with diagnosis and histological identification, (v) an available resource from pathology archives. Recently, FFPET-extracted DNA has been reported to be adequate for genotyping, and has been allowed for biomarker and functional genomics studies [23]–[29].

MALDI-TOF MS method, offering approximately 100% accuracy for SNP genotyping, is currently considered as a gold standard [29], [30]. Genotyping of FFPET-derived DNA by MALDI-TOF MS has been proven to be reliable and reproducible. Previously reports showed that there were no allelic frequency differences between FFPET-derived DNA and blood-derived DNA from the same individual through several methods including MALDI-TOF MS [24]–[26], [28]. Our efforts showed high success rates ranging between 96.29% and 100% (mean: 99.09%), which were in accordance with previous reported data [23], [24], [29].

SNPs are stably inherited, highly abundant and show diversity within and among populations, which are thought to be attractive biomarkers. However, the application of individual SNPs has been limited because they are low penetrance and their effects are relatively difficult to identify [5], [31]. Therefore, the importance of haplotype information has been increasing to link DNA sequence variation with disease [32]. Articles have reported that functional SNPs in HPSE were associated with heparanase expression differences and heparanase has been shown to be closely involved in the pathological process, progression and outcome of the disease [10], [22]. How to incorporate SNPs, however, in studies about gastric cancer predisposition and prognosis and how to determine the true associations are still challenging tasks.

There were no individual SNPs correlated to gastric cancer risk in our results. The associations between four individual SNPs (rs4328905, rs4693608, rs11099592 and rs6856901) and gastric cancer risk with 155 patients and 204 controls reported by Yue et al. [20] were in accordance with our results. Furthermore, all six common haplotypes had no significant differences in gastric cancer risk. This consistency showed SNPs in HPSE had no correlation to the incidence of gastric cancer in ethnic Han northern Chinese, not only from the perspective of individual SNPs, but also from the perspective of haplotypes.

In our present study, genotype rs4364254 TT was correlated to well cell differentiation. In addition, Ostrovsky et al. [22] found individuals with genotype TT possessed relatively high mRNA levels (P = 0.0029). However, there have been conflicting results reported as to associations between heparanase expression and histological differentiation. Endo K et al. [33] found that histological differentiation was worse in the heparanase mRNA-positive gastric cancer tissues (p<0.01). Chen JQ et al. [34] found that histologic differentiation was not related to heparanase mRNA expression in gastric cancer (P = 1.000). Takaomi Ohkawa et al. [35] demonstrated that heparanase expression was detected as stronger in well-differentiated cells (P = 0.0277), which is a finding that consistents with our results. Therefore, heparanase might be involved in cell differentiation, but the mechanisms are not clear at present.

In univariate analysis, patients carrying rs4693608 AA genotype had a poor survival (P = 0.049); in multivariate analysis, rs4693608 AA and rs4364254 TT both were significantly correlated with poor survival (P = 0.030 for rs4693608 AA and P = 0.013 for rs4364254 TT). Possibly, the absence of consensus in the univariate and multivariate analysis was due to the weak effect of the individual SNP, but when the individual SNP was considered together with the other SNP, Borrmann type, pT category, and pN category in multivariate analysis, it generated an influence on the prognosis. There is ample evidence to suggest that genetic factors contribute to the disease process in common complex trait diseases, but the effect of a single variant is probably small [36]. Besides, Ostrovsky et al. [37] provided a first evidence of correlation between functional SNPs rs4693608 and rs4364254 and risk of acute graft-versus-host disease (GVHD) development, and the rs4693608 was the most important. Their results were accordance to ours. In addition, Ostrovsky et al. [22] reported both rs4364254 TT genotype and also rs4693608 AA genotype were correlated to a relatively high mRNA level (P = 0.0029 and 0.004, respectively), which might partially explain a worse prognosis in patients with rs4693608 AA or rs4364254 TT in the present study. These observations were biologically plausible because overexpression of HPSE was closely associated with greater invasiveness of gastric cancer [38]–[40]. The present results demonstrated our presumption that SNPs were involved in the regulation of heparanase expression, thereby affecting invasion ability and survival in gastric cancer.

Though neither genotype rs11099592 CC nor rs4693608 AA showed a statistical difference in Borrmann type, haplotype CA composed with them did show a significant difference. Haplotype CA had a greater distribution in the group of Borrmann types 3 and 4 (P = 0.037). Perhaps patients with haplotype CA were more likely to develop a poorer general type. Besides, haplotype CA had a greater distribution in the N3 group (P = 0.046, compared to the N0 group). However, there were no significant distribution differences between the N2 and N0 groups and between the N1 and N0 groups. Perhaps there was an association between haplotype CA and greater numbers of lymph node metastases, but it needs further study. Moreover, patients carrying the CA haplotype also showed poor gastric cancer–specific survival, which was consistent with the differences in Borrmann type and numbers of lymph node metastases. Furthermore, Ostrovsky et al. [22] reported that rs11099592 CC genotype and rs4693608 AA genotype were correlated to high mRNA expression (P = 0.0167 and P = 0.004, respectively), which were in accordance with our results about haplotype CA. Perhaps the absolute risk associated with each of SNPs was low, but combined haplotype analysis may be more helpful in identifying individuals at high risk for progression of the disease. Perhaps it was specific haplotypes that play a significant role in gastric cancer invasion and metastasis, further affect prognosis.

A functional haplotype block composed of rs11099592 and rs4693608 was found in our results, which was associated with Borrmann type, pN category and prognosis of gastric cancer. On the one hand, SNP rs11099592 is an A-G substitution nonsynonymous SNP located in exon 8 and this alteration results in an arginine-to-lysine replacement at position 307, perhaps leading to a functional difference in the protein. On the other hand, SNP rs4693608 is located in intron 3 and showed a correlation to survival. Increasing amounts of evidence indicates that genomic variants in non-coding sequences might alter the expression of gene products by changing gene regulation, exon splicing, mRNA stability, cryptic splice sites activation and so on, which can therefore cause disease phenotypes. Besides, haplotypes may provide more relevant information than individual SNPs [7], [41]. Furthermore, whether gene transcription, maintaining of cellular differentiation and induction of an invasive metastatic phenotype are due to the direct interaction of heparanase with DNA is yet to be demonstrated.

Ostrovsky et al. [22] showed important association between combined genotypes for rs4693608 and rs4364254 SNPs and heparanase mRNA expression level. Furthermore, they divided all combined genotypes into three subgroups (LR-low expression, MR-intermediate expression, HR-high expression) according to heparanase mRNA expression level of each genotype, and they confirmed significant differences between three subgroups of combined genotypes carriers and mRNA levels. Besides, Ostrovsky et al. [37] first found correlations between combined genotypes for rs4693608 and rs4364254 SNPs and risk of acute GVHD development in their following study with this subgroups analysis method. It is an important and valuable method. Moreover, this method is useful for risk prediction associated with haplotype approach in following clinical practice. Our future study, which connected mRNA expression level to genotypes or haplotypes in HPSE of the Han population in northern China, would use this method.

Because the sample size of wild-type homozygote was relatively too small for stratified analysis on each genotype of all six SNPs investigated in our study, we could not show results of SNP analysis on each genotype, but we carried out analysis combined heterozygote with wild-type homozygote. It was a limitation of this study.

In conclusion, this study evaluated polymorphisms of the HPSE gene in gastric cancer with a MALDI-TOF MS method and archived FFPETs in a large northern Chinese case-controlled cohort. We found a functional haplotype block composed of rs11099592 and rs4693608, which was associated with Borrmann type, pN category and prognosis; and SNP rs4693608, which was included in the block, showed a correlation to survival. These results are supported by associations between SNPs in HPSE and mRNA expression levels reported previously by Ostrovsky et al. [22]. In addition, six individual SNPs and haplotypes were not correlated to gastric cancer risk. These results were consistent with our initial assumption that heparanase was involved in cancer invasion and metastasis and affected prognosis ultimately, but it was not involved in the incidence of cancer.

## Materials and Methods

### Sample collection

404 patients with histopathologically confirmed gastric cancer who had received radical surgery between January 1998 and December 2004 were consecutively selected. The patients were from northern China and were believed to be good representatives from this region. 404 normal gastric tissue samples were obtained from a segment of the resected specimens farthest from the tumor (>10 cm) and FFPETs were archived in the Surgical Oncology Department of the First Hospital of China Medical University in northern China. All samples were fixed and embedded under standard clinical histological conditions and were stored at room temperature. Paraffin sections of FFPETs were stained with hematoxylin and eosin (H&E) for pathological inspection to confirm the absence of tumorous tissue. The tumor histological grade was assessed according to World Health Organization criteria and tumors were staged using the 7th edition of the TNM staging of the International Union Against Cancer (UICC)/American Joint Committee on Cancer (AJCC) system (2010) based on postoperative pathologic examination of the specimens. Complete pathological data were obtained including age, gender, date of surgery, location of the primary tumor, histologic grade, venous invasion, lymphovascular invasion, depth of invasion, number of LNs retrieved, number of metastatic LNs, and number of tumor deposits retrieved. Those (i) with synchronous or metachronous malignant tumors, (ii) with distant metastasis found preoperatively, (iii) who underwent preoperative radiotherapy or chemotherapy, or (iv) with incomplete pathological data entries were excluded from this study. Follow-up was completed for the entire study population by January 2010. Two patients died in the postoperative period and 21 patients were lost during follow-up, therefore 381 patients were included in survival analysis. Median and mean follow-up periods were 90.0 months and 93.3±20.24 months (range: 61–136 months), respectively. The following data were obtained for all patients: date of death (if applicable), cause of death (if applicable), and date of follow-up. The primary endpoint was cause-specific survival duration from the date of gastric cancer diagnosis to the date of death. The 5-year survival rate of the 404 patients was 54.2%.

404 blood samples were obtained from cancer-free individuals who were randomly selected based on physical examinations during December 2009 to August 2011, as the control group, and this group was believed to be a good representation of the population in northern China region. The selection criteria included no individual history of cancer, frequency matching to cases on sex and age and individuals were unrelated ethnic Han Chinese. The samples (Ethylene Diamine Tetraacetic Acid [EDTA] anticoagulate) were stored at −20°C within 30–40 minutes, and then moved to a freezer at −80°C within 2 or 3 days after collection.

The study was approved by the Research Ethics Committee of China Medical University, China. Written informed consents were obtained from all patients before participating in the study.

### DNA extraction

Genomic DNA was extracted from FFPET samples in the case group. Sections with a thickness of 8 µm and a surface area of up to 250 mm^2^ were prepared with a microtome and DNA was isolated from 6 to 12 sections, depending on the tissue size and cell counts. The microtome was cleaned and blades were changed to avoid intersample contamination. DNA extraction from FFPETs was performed with QIAamp® DNA FFPE Tissue Kit (Qiagen, Hilden, Germany) [29], following the procedures described by the manufacturer, including (i) dissolve paraffin in xylene and remove, (ii) lyse sample under denaturing conditions with proteinase K, (iii) reverse the formalin crosslinking incubation at 90°C, (iv) bind DNA to the membrane and allow contaminants to flow through, (v) wash residual contaminants, and (vi) elute pure and concentrated DNA from the membrane (with tris-EDTA buffer [TE]). About 2–10 µg of DNA was recovered in 50 µl final solution and was stored at −80°C.

Genomic DNA was extracted from blood samples from the control group with the Universal Genomic DNA Extraction Kit Ver.3.0 (TAKARA) according to the manufacturer's instructions. About 2–6 µg of DNA was recovered in TE and was stored at −80°C.

### Selection of SNPs and genotyping

The study included six SNPs in HPSE, which were taken from the NCBI SNPs database (http://www.ncbi.nlm.nih.gov/snp) and the HapMap database (the Phase III database) (http://hapmap.ncbi.nlm.nih.gov/index.html.zh). These SNPs were mapped in HPSE gene (Figure 2). rs 11099592 was unique, not only with a minor allele frequency (MAF)>1%, but also as polymorphic in Han China Beijing (HCB) population among all coding region SNPs (cSNPs) in HPSE, which was registered in the databases. In addition, other five SNPs were located in intronic and 3′-UTR regions. Furthermore, other investigators have shown that rs11099592, rs4693608, and rs4364254 were correlated with heparanase mRNA expression [22]. Also, associations between individual SNPs or haplotypes in HPSE and susceptibility, clinicopathological parameters and prognosis of tumor reported in these articles was complex, but mostly concentrated on the six SNPs we selected [17]–[22].

SNPs were genotyped using the MALDI-TOF MS system (MassARRAY; Sequenom, San Diego, CA,USA) with primers and probes (Table S6) as previously described [29], [42]. To ensure the typing quality, 1% positive samples (YanHuang cell strain) were incorporated into every genotyping plate to validate the reliability of the primers and 1% negative samples (water with no DNA) to monitor contamination. 5% random samples were tested in duplicate by different persons and the reproducibility was 100%. The laboratory personnel were blinded to the sample arrangement during the process. There were six steps including PCR amplification, shrimp alkaline phosphatase treatment, base extension, salt removal with resin, SpectroCHIP dispensing (Sequenom, San Diego, CA,USA), and data acquisitions with MALDI-TOF MS according to Justenhoven et al. [43]. Finally, data analysis was performed using MassArray Typer Analyzer software 4.0.4.20 (Sequenom, San Diego, CA) [44].

### LD block determination and haplotype construction

Haploview 4.2 software was used to evaluate LD and construct haplotypes [31]. LD between the six SNPs used in haplotype analysis was measured by a pairwise D' statistic. The structure of the LD block was examined using the method of Gabriel et al. [45], using the 80% confidence bounds of D' to define sites of historical recombination between SNPs. Haplotypes were constructed from genotype data in the full-size case-control panel within blocks by using an accelerated expectation-maximization algorithm method [46]. Briefly, this method creates highly accurate population frequency estimates of the phased haplotypes based on the maximum likelihood as determined from unphased input [47].

### Statistical analysis

Statistical analysis was undertaken using the PASW Statistics 18.0 software (SPSS, Inc., Somers, NY, USA). A two-sided chi-square (χ2) test was used to estimate population distribution characteristics, compare differences in allelic and genotypic frequencies between cases and controls and assess associations between individual SNPs and clinicopathological parameters. A permutation procedure (1,000 tests) was used to correct the P value of single-locus association results. Odds ratios (OR) and confidence intervals (CI; 95%) were calculated by unconditional logistic regression to analyze the association between genotype frequencies and gastric cancer risk, and were adjusted for sex and age. Univariate and multivariate survival analysis were done with the log-rank test and Cox proportional hazards model using the clinicopathological parameters and SNPs. This resulted in the identification of covariates that significantly correlated with survival of the patients. Multivariate survival analysis was carried out by separately adding the SNP variables to all the clinicopathological parameters. A Kaplan-Meier method was used to map survival curves. The Haploview 4.2 software package was used to: estimate pair-wise linkage disequilibrium (LD), detect departure from the Hardy–Weinberg equilibrium, construct haplotype and calculate haplotype frequencies and estimate associations between haplotypes and gastric cancer risk. The Haplo.states software was used to assess associations between haplotypes and clinicopathologic features [31]. The THEsias software based on Cox proportional hazards survival regression in haplotype-based association analysis using the Stochastic-EM algorithm was used to produce survival analysis of haplotypes [48]. All tests were two-tailed and P<0.05 was considered statistically significant.

## Supporting Information

Figure S1 **Representative MALDI-TOF-MS profiles of each genotype of the six SNPs in HPSE.**
(TIF)

Figure S2 **Kaplan-Meier survival curve analysis with the different genotypes of rs4693602, rs6856901, rs4364254, rs11099592 and rs4328905.** Results show that accumulative survival rate of 381 cases with gastric cancer were associated with the different genotypes of rs4693602, rs6856901, rs4364254, rs11099592, and rs4328905 in HPSE.
(TIF)

Table S1 **Genotyping success rates of the six SNPs in HPSE.**
(DOC)

Table S2 **Associations between allele frequencies of the six SNPs in HPSE and the risk of gastric cancer (n = 404 for both case and control groups).**
(DOC)

Table S3 **Associations between genotype distributions of the six SNPs in HPSE and the risk of gastric cancer (n = 404 for both case and control groups).**
(DOC)

Table S4 **Associations between genotype distributions of the six SNPs in HPSE and clinicopathological parameters (n = 404).**
(DOC)

Table S5 **Associations between haplotype frequencies of the six SNPs in HPSE and the risk of gastric cancer (n = 404 for both case and control groups).**
(DOC)

Table S6 **Primer sequences used for genotyping the six SNPs in HPSE with the Sequenom platform.**
(DOC)
