# Supplementary figures and images for: Polymorphisms and a Haplotype in Heparanase Gene Associations with the Progression and Prognosis of Gastric Cancer in a Northern Chinese Population
Source: PLoS One. 2012 Jan 20;7(1):e30277. doi: 10.1371/journal.pone.0030277 (PMC3262795; doi:10.1371/journal.pone.0030277)

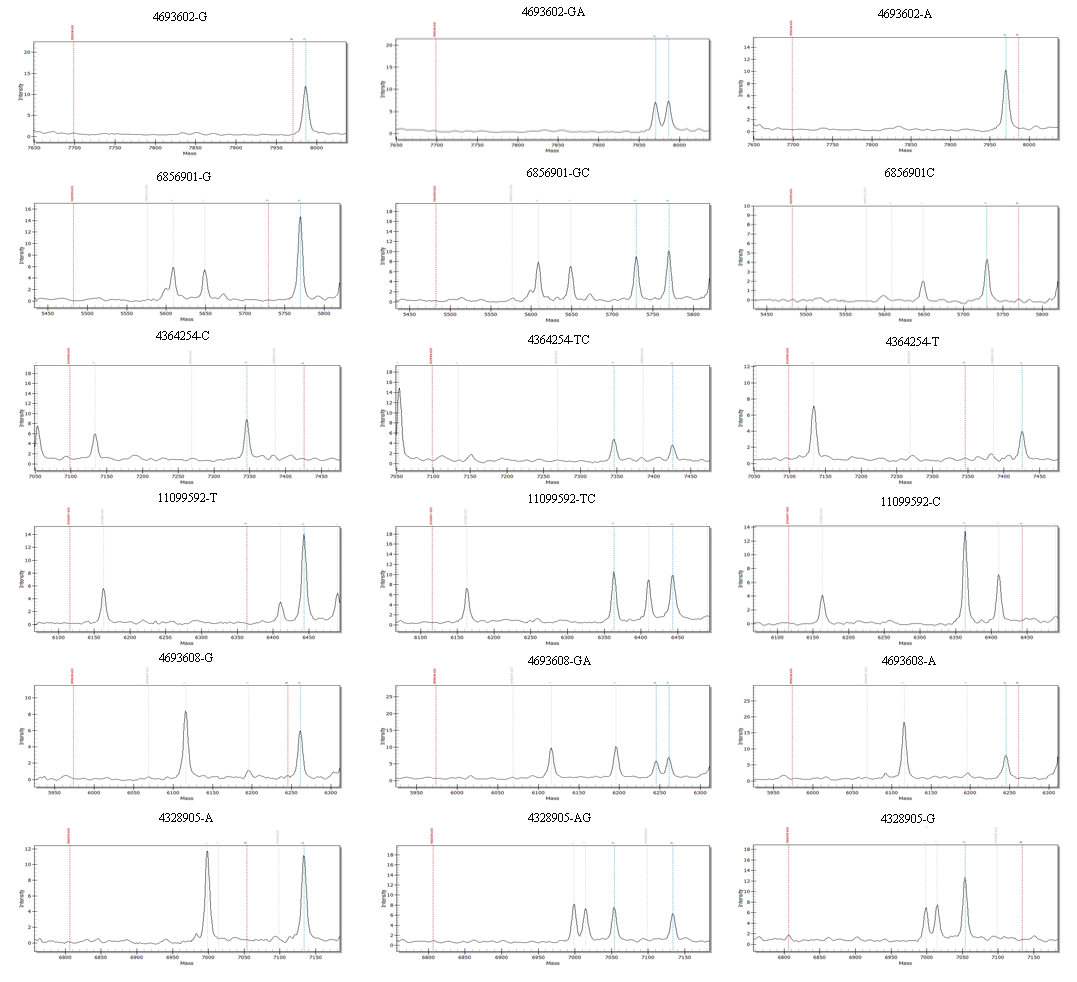

Supplement: Figure S1 — Representative MALDI-TOF-MS profiles of each genotype of the six SNPs in HPSE. (TIF) [file pone.0030277.s001.tif]

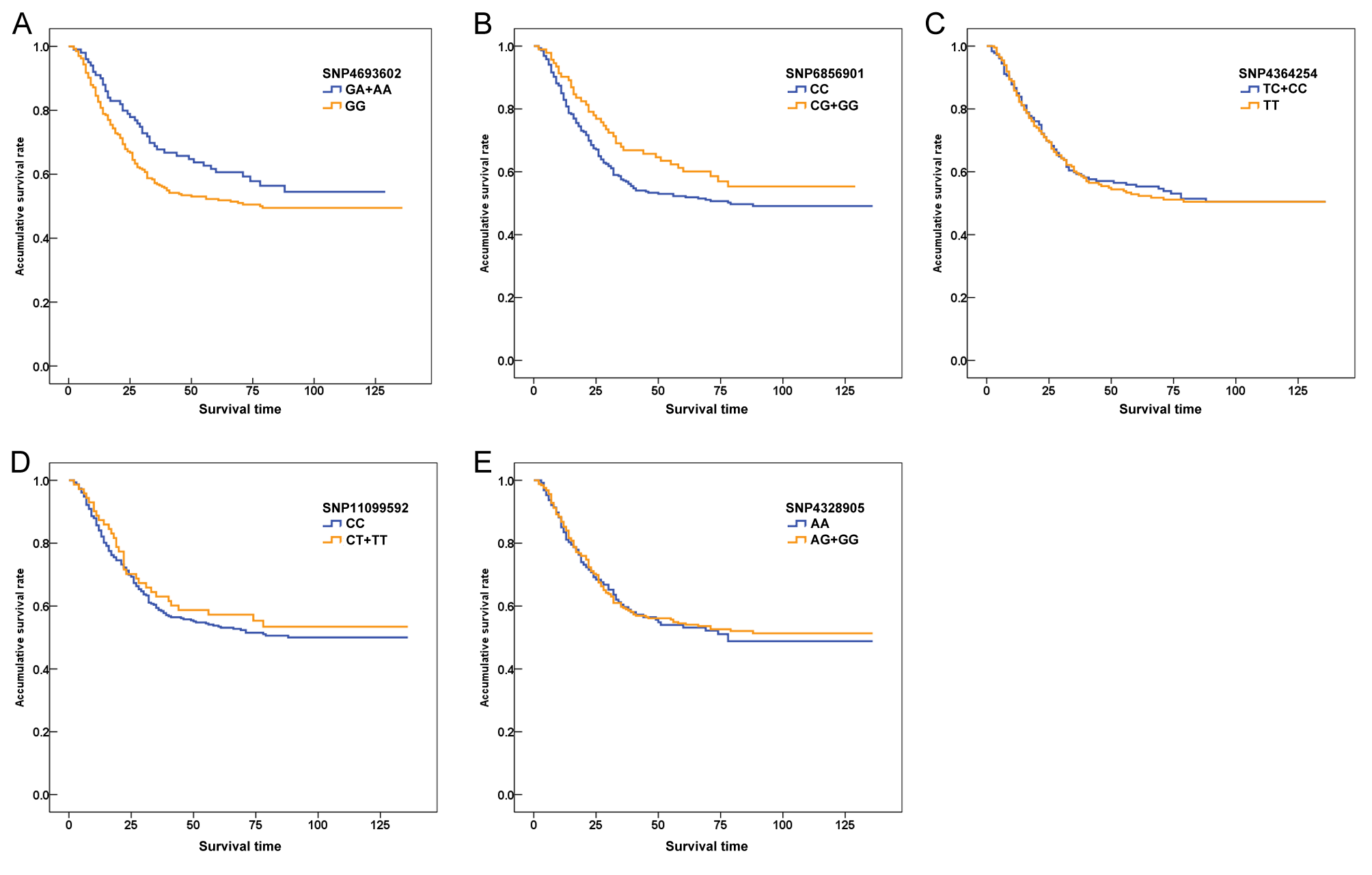

Supplement: Figure S2 — Kaplan-Meier survival curve analysis with the different genotypes of rs4693602, rs6856901, rs4364254, rs11099592 and rs4328905. Results show that accumulative survival rate of 381 cases with gastric cancer were associated with the different genotypes of rs4693602, rs6856901, rs4364254, rs11099592, and rs4328905 in HPSE. (TIF) [file pone.0030277.s002.tif]
