# Supplementary material for: Polymorphisms and a Haplotype in Heparanase Gene Associations with the Progression and Prognosis of Gastric Cancer in a Northern Chinese Population
Source: PLoS One. 2012 Jan 20;7(1):e30277. doi: 10.1371/journal.pone.0030277 (PMC3262795; doi:10.1371/journal.pone.0030277)
Supplement: Table S1 — Genotyping success rates of the six SNPs in HPSE. (DOC) [file pone.0030277.s003.doc]

**Table S1.** Genotyping success rates of the six SNPs in HPSE.

| SNP | Genotypes in FFPETs | | |  | Genotypes in Blood | | |
| --- | --- | --- | --- | --- | --- | --- | --- |
|  | Null (No.) | Total (No.) | Success rate (%) |  | Null (No.) | Total (No.) | Success rate (%) |
| rs4693602 | 15 | 404 | 96.29 |  | 2 | 404 | 99.50 |
| rs6856901 | 0 | 404 | 100.00 |  | 1 | 404 | 99.75 |
| rs4364254 | 2 | 404 | 99.50 |  | 1 | 404 | 99.75 |
| rs11099592 | 0 | 404 | 100.00 |  | 0 | 404 | 100.00 |
| rs4693608 | 3 | 404 | 99.26 |  | 1 | 404 | 99.75 |
| rs4328905 | 2 | 404 | 99.50 |  | 0 | 404 | 100.00 |
| Mean | 22 | 2424 | 99.09 |  | 5 | 2424 | 99.79 |
