# Supplementary material for: Polymorphisms and a Haplotype in Heparanase Gene Associations with the Progression and Prognosis of Gastric Cancer in a Northern Chinese Population
Source: PLoS One. 2012 Jan 20;7(1):e30277. doi: 10.1371/journal.pone.0030277 (PMC3262795; doi:10.1371/journal.pone.0030277)
Supplement: Table S2 — Associations between allele frequencies of the six SNPs in HPSE and the risk of gastric cancer (n = 404 for both case and control groups). (DOC) [file pone.0030277.s004.doc]

**Table S2.** Associations between allele frequencies of the six SNPs in HPSE and the risk of gastric cancer (n=404 for both case and control groups).

| SNP* | Position^§^ | Modification | Location | Risk allele | No.allele(%) | | P^‖^ | P^∫^ |
| --- | --- | --- | --- | --- | --- | --- | --- | --- |
|  |  |  |  |  | Patients | Controls |  |  |
| rs4693602 | 84213619 | G→A | 3’-UTR | A | 121(15.6) | 115(14.3) | 0.4587 | 0.9490 |
| rs6856901 | 84216368 | G→C | 3’-UTR | G | 108(13.4) | 95 (11.8) | 0.3386 | 0.8530 |
| rs4364254 | 84223713 | C→T | intron10 | T | 584(72.6) | 569(70.6) | 0.3637 | 0.8760 |
| rs11099592 | 84230619 | T→C  Arg→Lys | exon8 | C | 723(89.5) | 714(88.4) | 0.4756 | 0.9480 |
| rs4693608 | 84241357 | G→A | intron3 | A | 655(81.7) | 628(77.9) | 0.0608 | 0.2610 |
| rs4328905 | 84243549 | A→G | intron2 | A | 464(57.7) | 456(56.4) | 0.6049 | 0.9890 |

Abbreviation: UTR, untranslated region.

*According to National Center for Biotechnology Information SNP database rs number.

§Positions are from National Center for Biotechnology Information Build 37.2.

‖Two-sided χ^2^ test.

∫After 1,000 permutation tests.
