# Supplementary material for: Polymorphisms and a Haplotype in Heparanase Gene Associations with the Progression and Prognosis of Gastric Cancer in a Northern Chinese Population
Source: PLoS One. 2012 Jan 20;7(1):e30277. doi: 10.1371/journal.pone.0030277 (PMC3262795; doi:10.1371/journal.pone.0030277)
Supplement: Table S3 — Associations between genotype distributions of the six SNPs in HPSE and the risk of gastric cancer (n = 404 for both case and control groups). (DOC) [file pone.0030277.s005.doc]

**Table S3.** Associations between genotype distributions of the six SNPs in HPSE and the risk of gastric cancer (n=404 for both case and control groups).

| **Genotype** | **Patients** | **Controls** | **OR(95% CI)*** | **OR(95% CI)^☆^** | **P*** | **P^☆^** |
| --- | --- | --- | --- | --- | --- | --- |
|  | **No. (%)** | **No. (%)** |  |  |  |  |
| rs4693602 |  |  |  |  | 0.369 | 0.379 |
| GG | 281(72.2) | 294(73.1) | 1.00 | 1.00 |  |  |
| GA | 95(24.4) | 101(25.1) | 1.016(0.735-1.405) | 1.019(0.737-1.411) | 0.923 | 0.908 |
| AA | 13(3.3) | 7 (1.7) | 0.515(0.202--1.309) | 0.519(0.204-1.324) | 0.163 | 0.170 |
| GA+AA | 108(27.8) | 108(26.9) | 0.956(0.699-1.307) | 0.960(0.701-1.313) | 0.777 | 0.797 |
| rs6856901 |  |  |  |  | 0.605 | 0.631 |
| CC | 305(75.5) | 314(77.9) | 1.00 | 1.00 |  |  |
| CG | 90(22.3) | 83(20.6) | 1.383(0.472-4.054) | 1.382(0.471-4.052) | 0.554 | 0.556 |
| GG | 9(2.2) | 6(1.5) | 1.544(0.543-4.391) | 1.531(0.538-4.356) | 0.415 | 0.425 |
| CG+GG | 99(24.5) | 89(22.1) | 1.145(0.826-1.588) | 1.136(0.818-1.577) | 0.416 | 0.446 |
| rs4364254 |  |  |  |  | 0.269 | 0.257 |
| TT | 205(51.0) | 201(49.9) | 1.00 | 1.00 |  |  |
| TC | 174(43.3) | 167(41.4) | 0.979(0.734-1.306) | 0.984(0.737-1.313) | 0.884 | 0.912 |
| CC | 23(5.7) | 35(8.7) | 1.552(0.886-2.720) | 1.570(0.895-2.754) | 0.125 | 0.116 |
| TC+CC | 197(49.0) | 202(50.1) | 1.046(0.793-1.379) | 1.052(0.797-1.387) | 0.751 | 0.722 |
| rs11099592 |  |  |  |  | 0.634 | 0.624 |
| CC | 323(80.0) | 317(78.5) | 1.00 | 1.00 |  |  |
| CT | 77(19.1) | 80(19.8) | 1.059(0.747-1.501) | 1.059(0.747-1.502) | 0.749 | 0.748 |
| TT | 4(1.0) | 7(1.7) | 1.783(0.517-6.151) | 1.805(0.521-6.252) | 0.360 | 0.351 |
| CT+TT | 81(20.0) | 87(21.5) | 1.094(0.779-1.538) | 1.096(0.780-1.540) | 0.603 | 0.598 |
| rs4693608 |  |  |  |  | 0.178 | 0.177 |
| AA | 268(66.8) | 246(61.0) | 1.00 | 1.00 |  |  |
| AG | 119(29.7) | 136(33.7) | 1.245(0.922-1.682) | 1.249(0.924-1.687) | 0.153 | 0.148 |
| GG | 14(3.5) | 21(5.2) | 1.634(0.813-3.284) | 1.626(0.809-3.271) | 0.168 | 0.173 |
| AG+GG | 133(33.2) | 157(39.0) | 1.286(0.964-1.716) | 1.289(0.965-1.720) | 0.088 | 0.085 |
| rs4328905 |  |  |  |  | 0.691 | 0.700 |
| AA | 135(33.6) | 125(30.9) | 1.00 | 1.00 |  |  |
| AG | 194(48.3) | 206(51.0) | 1.147(0.839-1.567) | 1.144(0.837-1.560) | 0.390 | 0.399 |
| GG | 73(18.2) | 73(18.1) | 1.080(0.720-1.620) | 1.018(0.718-1.617) | 0.710 | 0.717 |
| AG+GG | 267(66.4) | 279(69.1) | 1.129(0.840-1.517) | 1.126(0.838-1.513) | 0.423 | 0.432 |

*Data were calculated by unconditional logistic regression.

^☆^Data were calculated by unconditional logistic regression, and adjusted for sex, age.

Abbreviation: OR, odds ratio; CI, confidence interval.
