# Supplementary material for: Polymorphisms and a Haplotype in Heparanase Gene Associations with the Progression and Prognosis of Gastric Cancer in a Northern Chinese Population
Source: PLoS One. 2012 Jan 20;7(1):e30277. doi: 10.1371/journal.pone.0030277 (PMC3262795; doi:10.1371/journal.pone.0030277)
Supplement: Table S4 — Associations between genotype distributions of the six SNPs in HPSE and clinicopathological parameters (n = 404). (DOC) [file pone.0030277.s006.doc]

**Table S4. Associations between genotype distributions of the six SNPs in HPSE and clinicopathological parameters (n=404).**

| **Polymorphisms/genotypes*** | | | | | | | | | | | | | |
| --- | --- | --- | --- | --- | --- | --- | --- | --- | --- | --- | --- | --- | --- |
|  | rs4693602 | | rs6856901 | | rs4364254 | | rs11099592 | | rs4693608 | | rs4328905 | | |
|  | GG | GA/AA | CC | CG/GG | TT | TC/CC | CC | CT/TT | AA | AG/GG | AA | AG | GG |
| Borrmann type |  |  |  |  |  |  |  |  |  |  |  |  |  |
| Borr1+2 | 72 | 36 | 77 | 32 | 56 | 53 | 84 | 25 | 66 | 43 | 36 | 54 | 18 |
| Borr3+4 | 209 | 72 | 228 | 67 | 141 | 152 | 239 | 56 | 202 | 90 | 99 | 140 | 55 |
| *P* | 0.128 |  | 0.168 |  | 0.562 |  | 0.378 |  | 0.103 |  | 0.871 |  |  |
| Histologic grade |  |  |  |  |  |  |  |  |  |  |  |  |  |
| Well | 64 | 25 | 71 | 23 | 59 | 35 | 73 | 21 | 61 | 33 | 35 | 46 | 13 |
| Poor | 217 | 83 | 234 | 76 | 138 | 170 | 250 | 60 | 207 | 100 | 100 | 148 | 60 |
| *P* | 0.938 |  | 0.992 |  | **0.002^☆^** |  | 0.527 |  | 0.648 |  | 0.414 |  |  |
| pT category |  |  |  |  |  |  |  |  |  |  |  |  |  |
| T1 | 37 | 22 | 40 | 19 | 32 | 27 | 44 | 15 | 35 | 24 | 21 | 31 | 7 |
| T2 | 53 | 14 | 56 | 14 | 32 | 37 | 53 | 17 | 45 | 25 | 25 | 30 | 15 |
| T3 | 130 | 52 | 146 | 47 | 95 | 98 | 158 | 35 | 127 | 63 | 69 | 91 | 33 |
| T4 | 61 | 20 | 63 | 19 | 38 | 43 | 68 | 14 | 61 | 21 | 20 | 42 | 18 |
| *P* | 0.196 |  | 0.434 |  | 0.805 |  | 0.433 |  | 0.282 |  | 0.442 |  |  |
| pN category |  |  |  |  |  |  |  |  |  |  |  |  |  |
| N0 | 81 | 43 | 88 | 39 | 68 | 58 | 103 | 24 | 83 | 44 | 43 | 61 | 23 |
| N1 | 38 | 9 | 42 | 8 | 24 | 26 | 41 | 9 | 31 | 18 | 17 | 25 | 7 |
| N2 | 64 | 22 | 70 | 20 | 44 | 46 | 72 | 18 | 58 | 31 | 34 | 40 | 16 |
| N3 | 98 | 34 | 105 | 32 | 61 | 75 | 107 | 30 | 96 | 40 | 41 | 68 | 27 |
| *P* | 0.160 |  | 0.176 |  | 0.532 |  | 0.913 |  | 0.710 |  | 0.917 |  |  |
| Venous invasion |  |  |  |  |  |  |  |  |  |  |  |  |  |
| Negative | 279 | 107 | 303 | 97 | 194 | 204 | 319 | 81 | 264 | 133 | 134 | 193 | 71 |
| Positive | 2 | 1 | 2 | 2 | 3 | 1 | 4 | 0 | 4 | 0 | 1 | 1 | 2 |
| *P* | 1.000 |  | 0.253 |  | 0.296 |  | 0.588 |  | 0.307 |  | 0.247 |  |  |
| Lymphovascular invasion |  |  |  |  |  |  |  |  |  |  |  |  |  |
| Negative | 205 | 85 | 223 | 80 | 150 | 152 | 247 | 56 | 207 | 61 | 106 | 145 | 51 |
| Positive | 76 | 23 | 82 | 19 | 47 | 53 | 76 | 25 | 94 | 39 | 29 | 49 | 22 |
| *P* | 0.244 |  | 0.125 |  | 0.644 |  | 0.173 |  | 0.153 |  | 0.381 |  |  |
| TNM stage |  |  |  |  |  |  |  |  |  |  |  |  |  |
| I | 57 | 25 | 62 | 23 | 43 | 41 | 68 | 17 | 56 | 29 | 29 | 42 | 14 |
| II | 73 | 31 | 80 | 27 | 57 | 50 | 85 | 22 | 65 | 41 | 35 | 51 | 21 |
| III | 151 | 52 | 163 | 49 | 97 | 114 | 170 | 42 | 147 | 63 | 71 | 101 | 38 |
| *P* | 0.610 |  | 0.759 |  | 0.424 |  | 0.988 |  | 0.296 |  | 0.988 |  |  |

*Two-sided χ^2^ test.

**^☆^**Statistically signiﬁcant (P<0.05); OR=0.482; 95% CI: 0.300-0.774 comparison to the genotype TC/CC.
