# Supplementary material for: Polymorphisms and a Haplotype in Heparanase Gene Associations with the Progression and Prognosis of Gastric Cancer in a Northern Chinese Population
Source: PLoS One. 2012 Jan 20;7(1):e30277. doi: 10.1371/journal.pone.0030277 (PMC3262795; doi:10.1371/journal.pone.0030277)
Supplement: Table S5 — Associations between haplotype frequencies of the six SNPs in HPSE and the risk of gastric cancer (n = 404 for both case and control groups). (DOC) [file pone.0030277.s007.doc]

**Table S5. Associations between haplotype frequencies of the six SNPs in HPSE and the risk of gastric cancer (n=404 for both case and control groups).**

| Haplotype | All subjects | Patients | Controls | P* | P^☆^ |
| --- | --- | --- | --- | --- | --- |
| Block 1 |  |  |  |  |  |
| GC | 0.850 | 0.842 | 0.848 | 0.391 | 0.9700 |
| AG | 0.126 | 0.134 | 0.131 | 0.3386 | 0.9520 |
| AC | 0.025 | 0.024 | 0.021 | 0.945 | 1.0000 |
| overall |  |  |  | 0.6397 | 0.623 |
| Block 2 |  |  |  |  |  |
| CA | 0.798 | 0.817 | 0.776 | 0.0579 | 0.3440 |
| TG | 0.111 | 0.105 | 0.123 | 0.4756 | 0.9840 |
| CG | 0.091 | 0.077 | 0.101 | 0.0616 | 0.3550 |
| Overall |  |  |  | 0.1152 | 0.116 |

*Two-sided χ^2^ test, each haplotype compared with all other haplotypes.

^☆^After 1,000 permutation tests.
