# Supplementary material for: Polymorphisms and a Haplotype in Heparanase Gene Associations with the Progression and Prognosis of Gastric Cancer in a Northern Chinese Population
Source: PLoS One. 2012 Jan 20;7(1):e30277. doi: 10.1371/journal.pone.0030277 (PMC3262795; doi:10.1371/journal.pone.0030277)
Supplement: Table S6 — Primer sequences used for genotyping the six SNPs in HPSE with the Sequenom platform. (DOC) [file pone.0030277.s008.doc]

**Table S6.** Primer sequences used for genotyping the six SNPs in HPSE with the Sequenom platform.

| **Identity** | **Forward primers** | **Reverse Primers** | **Extension primers** |
| --- | --- | --- | --- |
| rs4693602 | ACGTTGGATGGTCTGTAGGCTTTACATTAC | ACGTTGGATGTTTTCACATTTGAAATAAAG | AAAAGAATAGTCATACAATATCAAG |
| rs6856901 | ACGTTGGATGTAGCAGTGTTCTACCTAGCG | ACGTTGGATGTGGGTGCTTGAGAGACACTG | TGCTAAATCTAGCACTGA |
| rs4364254 | ACGTTGGATGTCTGTCAAGAATGATCAGAG | ACGTTGGATGAGTTTGGCTTTGAGCTTTGC | AGAGTTTAAGTATTCTTGGTTAT |
| rs11099592 | ACGTTGGATGAAATGTCCAATACATCAGGG | ACGTTGGATGGCTACTATTTGAATGGACGG | CAGGGTTTAGAAAATCTTCC |
| rs4693608 | ACGTTGGATGTTTCCTCTTGCCATCATGGG | ACGTTGGATGGCTCTGATGGTACCAAATTC | GGTAGGATGGGTGGGAACC |
| rs4328905 | ACGTTGGATGGTTTGACTAGATTGTCTTGG | ACGTTGGATGCAAGGTACGAAGCTTTGGAG | GACTAGATTGTCTTGGAAAAAA |
